# A Multi-Wavelength Opto-Electronic Patch Sensor to Effectively Detect Physiological Changes against Human Skin Types

**DOI:** 10.3390/bios7020022

**Published:** 2017-06-21

**Authors:** Liangwen Yan, Sijung Hu, Abdullah Alzahrani, Samah Alharbi, Panagiotis Blanos

**Affiliations:** 1 School of Mechatronic Engineering and Automation, Shanghai University, Shanghai 200072, China; lw_yan@staff.shu.edu.cn; 2 School of Electronic, Electrical and Systems Engineering, Loughborough University, Ashby Road, Loughborough, Leicestershire LE11 3TU, UK; A.Alzahrani@lboro.ac.uk (A.A.); S.M.Alharbi@lboro.ac.uk (S.A.); P.Blanos@lboro.ac.uk (P.B.); 3 College of Engineering, Taif University, Airport Road, Al Hawiyah Area 888, Taif 26571, Saudi Arabia

**Keywords:** multi-wavelength, auto adaptive adjustment, opto-electronic patch sensor (OEPS), skin pigments, Von Luschan’s chromatic scale (VLCS)

## Abstract

Different skin pigments among various ethnic group people have an impact on spectrometric illumination on skin surface. To effectively capture photoplethysmographic (PPG) signals, a multi-wavelength opto-electronic patch sensor (OEPS) together with a schematic architecture of electronics were developed to overcome the drawback of present PPG sensor. To perform a better in vivo physiological measurement against skin pigments, optimal illuminations in OEPS, whose wavelength is compatible with a specific skin type, were optimized to capture a reliable physiological sign of heart rate (HR). A protocol was designed to investigate an impact of five skin types in compliance with Von Luschan’s chromatic scale. Thirty-three healthy male subjects between the ages of 18 and 41 were involved in the protocol implemented by means of the OEPS system. The results show that there is no significant difference (*p:* 0.09, *F* = 3.0) in five group tests with the skin types across various activities throughout a series of consistent measurements. The outcome of the present study demonstrates that the OEPS, with its multi-wavelength illumination characteristics, could open a path in multiple applications of different ethnic groups with cost-effective health monitoring.

## 1. Introduction

Pigments in various human skin types could affect signal quality in photoplethysmography (PPG) and could even make physiological measurements invalid. The quality of PPG-based in vivo physiological monitoring is attributed to melanin concentration of skin and its related pigments of skin types [1]. Thereby, melanin in human skin is known to highly attenuate incident light with relevant wavelength illuminations [2]. Some researchers reported anecdotal errors (3–5%) in black skin [3]. In addition, modelling and simulations were conducted and errors due to various pigments were reported [3,4]. Ink on skin and nail polish could cause errors during measurements [5]. Four different wavelength illuminations have attempted to optimize the measurements of heart rate (HR) by using a customized PPG setup on 23 healthy subjects with various skin types on PPG system [6]. The results showed that the 520 nm wavelength (green) illumination provided a significantly greater (*p* < 0.001) ability to detect HR. Increasing levels of melanin, or darker skin type (Type V) showed a decreasing trend with insignificant change (*p* < 0.067). Moreover, it has been widely confirmed that other forms of medium or material, i.e., fingerprinting ink and henna, could bring some issues to optical absorption and thus produce errors during the measurements. Some kinds of wrist tattoos could also interfere with PPG sensors on the back of smart watches, resulting in errant watch functionalities with users [7].

The skin of the human body is comprised of three main layers: epidermis, dermis and subcutaneous. Melanin only plays an essential role in skin color and is responsible for a wide variety of skin color complexions. At present, Von Luschan’s chromatic scale (VLCS) and Fitzpatrick scale (FPS) are applied in skin classification. Usually, VLCS [8] is applied to establish racial classifications of populations based upon skin colors. In contrast, FPS is considered [9] for classification of individual skin type, as introduced in 1975, to describe sun-tanning behavior. FPS distinguishes several separate skin tones that culturally fall under “white,” or Caucasian, and does not make an adequate distinction on the darker side of human skin color gradient [10]. Hence, VLCS was applied in this study to better describe the actual varieties of skin colors within these ethnic group people. The majority of skin types, in corresponding regions, are defined in Table 1.

**Figure 1:**
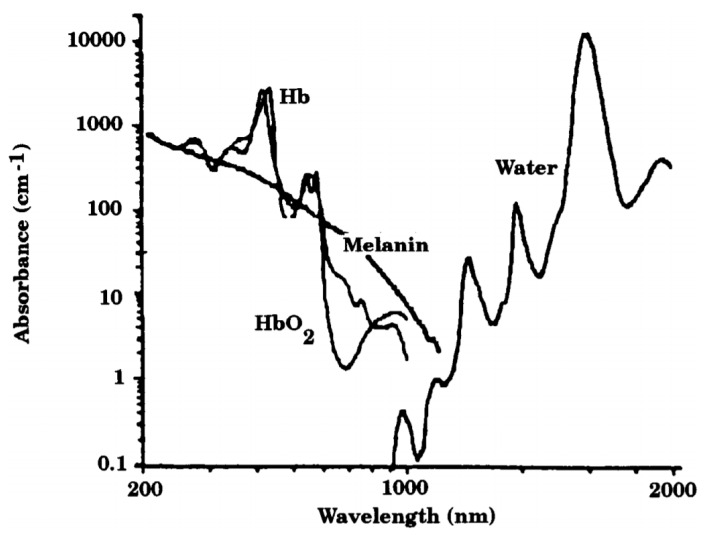
Skin tissue spectral window [12].

Meanwhile, it would be useful to reveal human tissue optical properties in order to better understand the interaction of illumination to tissue with physiological variations. Skin absorption rates are usually various when the skin is illuminated by different wavelength illuminations. Figure 1 illustrates an optical window of human tissue in which the illumination is greatly absorbed by melanin within the ultraviolet band (10–400 nm), whereas the water substance is strongly absorbed in the range of longer wavelength. Generally, the optical window (405–1064 nm) is often chosen for opto-physiological measurements [11], such as a typical application of pulse oximeter.

In addition, the illumination wavelength of PPG is usually determined based on the absorption of oxyhemoglobin (Hb) and deoxyhemoglobin (HbO_2_) associated with blood volume changes in peripheral and capillary arterial vessels. Namely, the illumination associated with specific wavelengths to skin with rich peripheral blood vessels are preferable to surface skin layers where there is no arterial blood or scattering. Table 2 shows the wavelengths of optical radiation reaching a certain penetration depth.

Various skin pigments are highly correlated with spectrometric illumination on skin surface, thus impacting the quality of the PPG measurements as shown in Table 2. In addition, different optical radiation wavelengths can reach different penetration depths of skin tissue. Hence, this study aims to investigate measurement effectiveness of multi-wavelength illumination based on opto-electronic patch sensor (OEPS) against skin pigments using new electronic architecture with auto adaptive adjustment of signals.

## 2. Materials and Methods

### 2.1. OEPS Configuration

**Figure 2:**
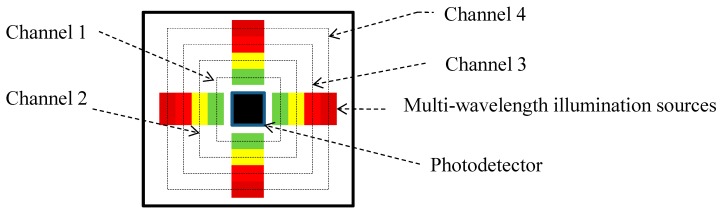
Schematic diagram of opto-electronic patch sensor (OEPS). The multi-wavelength illumination sources of OEPS comprises (1) channel 1 with four green light-emitting diodes (LEDs); (2) channel 2 with four yellow LEDs; (3) channel 3 with four red LEDs and (4) channel 4 with four IR LEDs.

An OEPS (physical size: 18 mm × 18 mm × 0.1 mm), as shown in Figure 2, consisted of (1) 16 light-emitting diodes (LEDs, JMSIENNA Co., Ltd., TouFen, Taiwan) as illumination sources [14]. Their peak wavelengths were 525 nm (green), 590 nm (yellow), 650 nm (red) and 870 nm (IR) respectively, and (2) A Si-photodiode (PD) with a large active area (1.69 mm^2^, S10625, Hamamatsu photonics K. K., Hamamatsu City, Japan) as a photodetector. The PD and LEDs were mounted side-by-side (reflection mode PPG). The PCB routing and footprints were allocated by PADS (Pads PCB PADS Standard, Wilsonville, OR, USA). A layer of clear epoxy medical adhesive was also used to protect the optical components.

### 2.2. Electronic Composition

**Figure 3:**
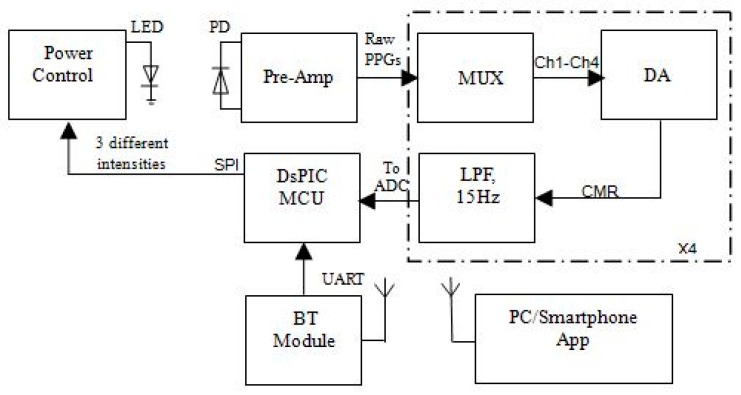
Block diagram of the OEPS circuit with six individual functional modules: (1) DsPIC MCU: microcontroller (dsPIC33FJ64GS610); (2) LPF: low-pass filter (fc = 15 Hz); (3) DA: differential amplifier; (4) MUX: multiplexer; (5) Pre-Amp: pre-amplifiers; and (6) BT Module: Bluetooth module.

The OEPS system, with a microcontroller DsPIC (dsPIC33FJ64GS610, Microchip Technology Inc., Chandler, AZ, USA), has multi-functional control and plays an interfering role. Figure 3 shows a block diagram of OEPS to multiplex and change LED light intensity through a power control. A Bluetooth module (CC2541F256, Texas Instruments Inc., Boulevard Dallas, TX, USA) driven by DsPIC processor. A pre-amplifier (Pre-Amp), a Multiplexer (MUX), a differential amplifier (DA) and a low-pass filter (LPF) are the main parts of analogue front-end (AFE) electronics in the system.

Since an alternative component (AC) is 2–5% of the static component DC [15], thus the raw signal from the PD needs to be amplified by the Pre-Amp. The MUX is also used as a demultiplexer, as the output of the PD is a pulse train of multi-wavelength illuminations. The common mode rejection of the differential amplifier (DA) is driven to a LPF to eliminate a high frequency (HF), such as power supply frequency and electromagnetic. In addition, the LPF is playing an important role for preventing the aliasing components from being sampled. Then the signal from the LPF is moved to the DsPIC processor, and is then converted to a digital form through an analogue-to-digital convert (ADC). The wireless transmission is achieved by a Bluetooth module that receives the digital PPG signals from the central processor, through the USART protocol, to then send the signals to a PC and/or a smartphone device.

### 2.3. Execution of OEPS System

Three main functionalities operating the OEPS system are outlined as follows:

#### (1) Time sequence of multiplexing LED and demultiplexing signals from the PD.

**Figure 4:**
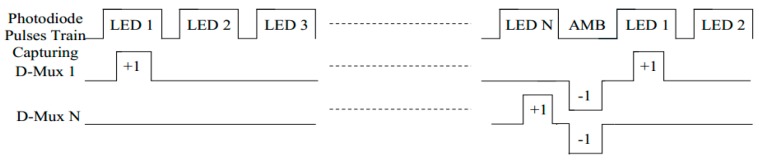
Schematic diagram of time switching in multiplexed illumination of LEDs and demultiplexed signals from a photodiode.

A time multiplexing algorithm is employed in DsPIC to asynchronously switch the LEDs to “ON” and “OFF”. The four channel illuminations (green, yellow, red and infrared LEDs) are multiplexed at the frequency of 1 kHz. Figure 4 is a schematic diagram of time switching for multiplexed illumination of four LED channels and demultiplexing of the signals from the photodiode. The time sequence is implemented a timer interrupt for accurate performance. DsPIC is used to switch the LEDs, alternating them between two states (“OFF”, “ON”) and provides only a single wavelength LED illumination at a time.

On the other hand, the switching time between these illumination sources is crucial to distinguish between different LED wavelengths, otherwise the PD produces only interference pulse trains with a combination of multi-wavelength illuminations. The output of PD requires demultiplexing where one specific wavelength illumination is extracted from these combined signals at a certain time. After cancelling the effect of ambient light and common mode rejection, the signals from these individual LED illuminations are acquired respectively and passed through a LPF (fc = 15 Hz) where they are converted to digital signals inside the DsPIC processor.

#### (2) Auto adaption for LED illumination intensity.

Different user skin color pigmentation as well as skin and fat thickness lead to various light absorptions. A constant intensity, i.e., driving LEDs in conventional methods [16], could easily cause an unexpected saturated response from the PD or limited signal amplitude. When the intensity of LEDs illumination is unexceptionally adjusted with different types of skin pigments, raw signals from the PD could sometimes be saturated. Hence, AC amplitude that extracted from the raw signals, is insignificant with a highly limited dynamic range. In this study, the auto-intensity calibration was fulfilled with these requirements. Three DAC values of LED illumination intensity are pre-set as (1) desired signal level, (2) lower threshold, and (3) upper threshold. When a raw signal from any illumination channel is either smaller than the lower threshold or larger than the upper threshold, an optimal adaptation of illumination current is implemented as designated. An optimal adaptation period of two to three seconds sets the LED current to an optimal value resulting in the received signal approaching the desired value. The auto-control procedure is used to automatically adjust the LED currents following a large step-change in the received signals.

**Figure 5:**
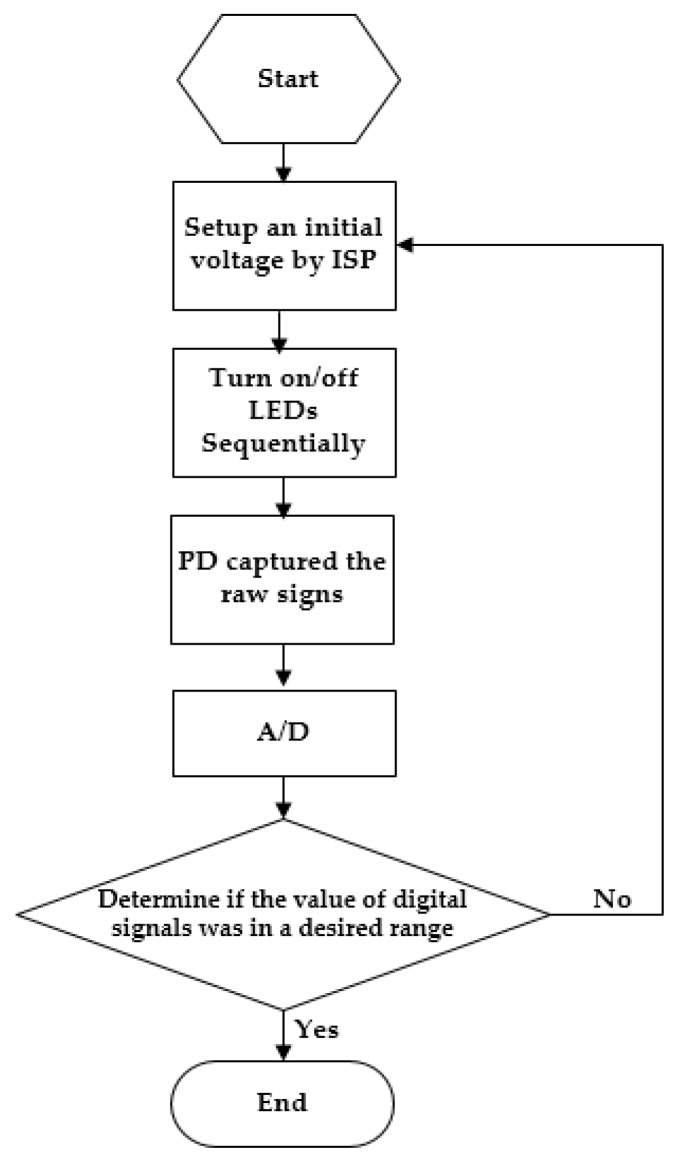
The flowchart against saturated signal. ISP: interface serial port; PD: Si-photodiode.

For the OEPS to work accurately under different conditions, it is necessary to optimize the desired signal level and/or the thresholds as shown in Figure 5. While the system is initialized, the LED voltage is powered by the microcontroller through an interface serial port (ISP), and then a series of time switch for multiplexing LED illuminations is established. The raw signal is captured by the PD to be then converted to a digital signal via ADC. The output of the analog signal circuit is connected to the ADC channel of the dsPIC. One ADC sample is taken during each LED’s ON-time period, the adjust DAC values change accordingly to calibrate the LED intensity and keep the output signal level within the microcontroller’s ADC range. Finally, an optimal value between the thresholds of saturated and digital outputs is chosen. Once the value is set to a desired range, the programmed loop is ended and sets the value as an output voltage of LEDs. Otherwise, the voltage flowchart is repeated till the desired value is attained.

#### (3) OEPS measurement procedures.

**Figure 6:**
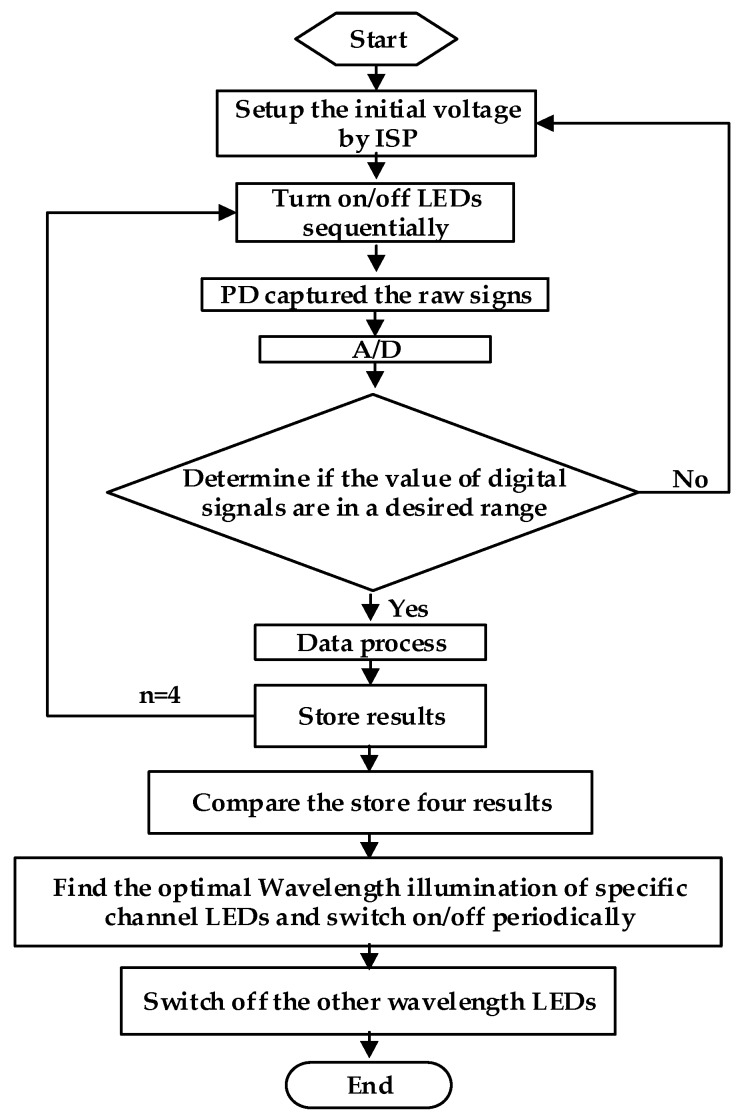
The flowchart against the effect of skin pigments.

Figure 6 is the outline of the OEPS measurement procedures against the effect of skin pigments. When one of the LED channels is lighted on, the raw signal is captured by the PD to be then digitized, and stored as a record or dataset. Since there are four LED channels with four individual wavelength illuminations, the same operation is executed four times, and four records of each individual channels are stored simultaneously. After these four records are compared, the optimal wavelength illumination of a specific LED channel is implemented in the measurement against skin pigments.

### 2.4. Experimental Protocol

The OEPS system against these skin types was performed by evaluating four different types of physical activity [17]: (1) resting, (2) walking at 3 km/h, (3) jogging at 6 km/h, and (4) running at 9 km/h. Thirty-three male subjects between the ages of 18 and 41 participated in the experimental protocol with the approval of the Loughborough University Ethics Committee. Prior to recording, each subject’s body mass index (BMI: 18.7–33.3) and blood pressure (108/62–147/79) were documented, as well as room temperature (23–26 °C) and humidity (22–36%). The 33 subjects were also divided into four groups according to their skin type. They were asked to perform, having the OEPS attached to their palm, a variety of designated similar activities within a 60 s duration for each activity i.e., resting, walking, jogging and running. Whilst individual recordings for each subject were taken, the signal processing for heart rate (HR) was performed by MATLAB (MathWorks Inc., Natick, MA, USA) via these two procedures: (1) filtration condition of band-pass filter, LPF and high-pass filter (HPF) and (2) HR readings obtained from Fast Fourier Transform (FFT). The signal processing for the HR was carried out every 15 second sampling. Specifically, at resting, the HR values were shown on the resting on electrocardiogram (ECG) screen in real time and they were recorded every 5 seconds, then their mean HR value was calculated to be compared with the HR extracted from PPG signals.

In order to identify the influence of different skin types on the HR, the average value of HR and a standard deviation SD were calculated referring to the five different skin type groups during the four types of exercises. A two-way analysis of variance (ANOVA) was executed to determine whether there were any statistically significant differences between the mean values of the skin type group to the four groups of exercises, i.e. resting, walking, jogging and running.

## 3. Results

**Figure 7:**
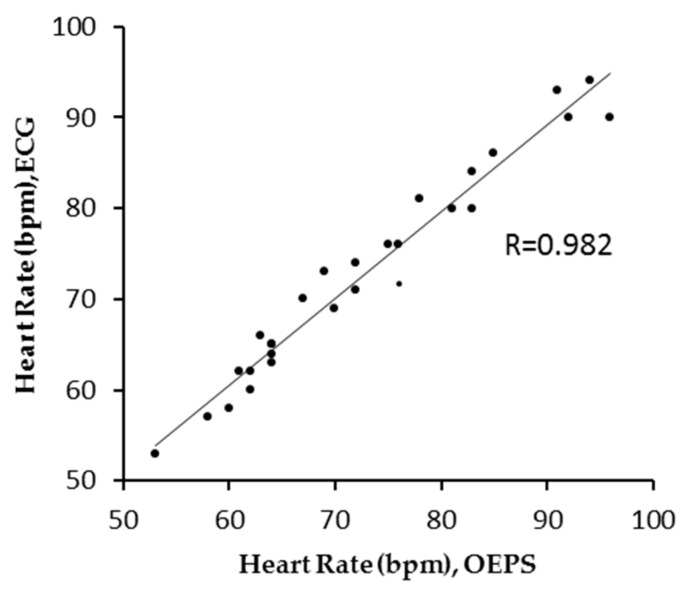
HR Correlation between OEPS and ECG with (*r* = 0.982) during rest mode.

The evaluation of the HR readings between two measurement techniques, i.e., OEPS and three-lead resting ECG (AT-10 Plus, Schiller UK Ltd., Bellshill, UK) were performed using unpaired *t*-test. Pearson’s correlation analysis was also used to correlate quantitative variables (*r* ≥ 0.98), as an indicator of two techniques evolving in parallel, as shown in Figure 7. The test showed that there was no significant difference since there is a probability value of (*p* = 0.99) with a condition of (*p* < 0.05).

**Figure 8:**
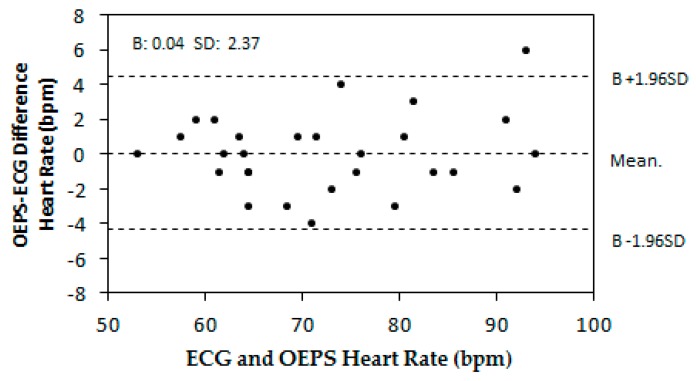
Differences in heart rate (HR) outputs recorded at resting using Bland–Altman plot. The acceptable range: B ± 1.96SD.

The Bland–Altman method was used to compare the values of the HR obtained between the OEPS and the commercial ECG devices. Figure 8 indicates the bias B: 0.04 bpm, standard deviation SD = 2.37 bpm, lower and upper limits of agreement, −4.60 bpm and +4.68 bpm respectively.

Thirty-three healthy male subjects, with five different skin types divided in four ethnic groups, were selected to participate in the implementation of the experimental protocol. The average value range (AVR, bpm) and the standard deviation range (SDR, bpm) of these five skin types of subjects, during the four activities, are summarized in Table 3. In resting, the AVRs of HR were from 57 to 94 and SDRs were from 1.0 to 5.9. Whilst running, the AVRs of HR were increased from 98 to 170 and the SDRs from 3.1 to 21.9 bpm. The largest value of SDR was at 21.9 bpm.

**Figure 9:**
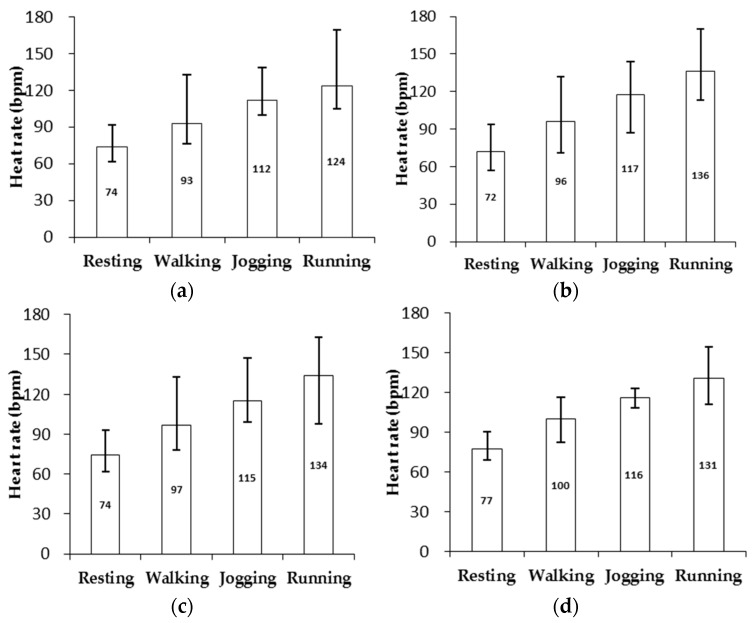
HR measurement results of different skin types at four types of exercises, i.e., resting, walking, jogging and running. (**a**) Mean HR and range (skin type I & II); (**b**) Mean HR and range (skin type III); (**c**) Mean HR and range (skin type IV); (**d**) Mean HR and range (skin type V).

The mean HRs and the lower and upper limits of HR in the four exercise conditions (resting, walking, jogging and running) were obtained referring to the skin types as shown in Figure 9.

The results through the two-way ANOVA execution displays that *p*1 ≈ 0 (for activities) and *p*2 = 0.09 (for skin types) as obtained from the list in Table 4.

**Figure 10:**
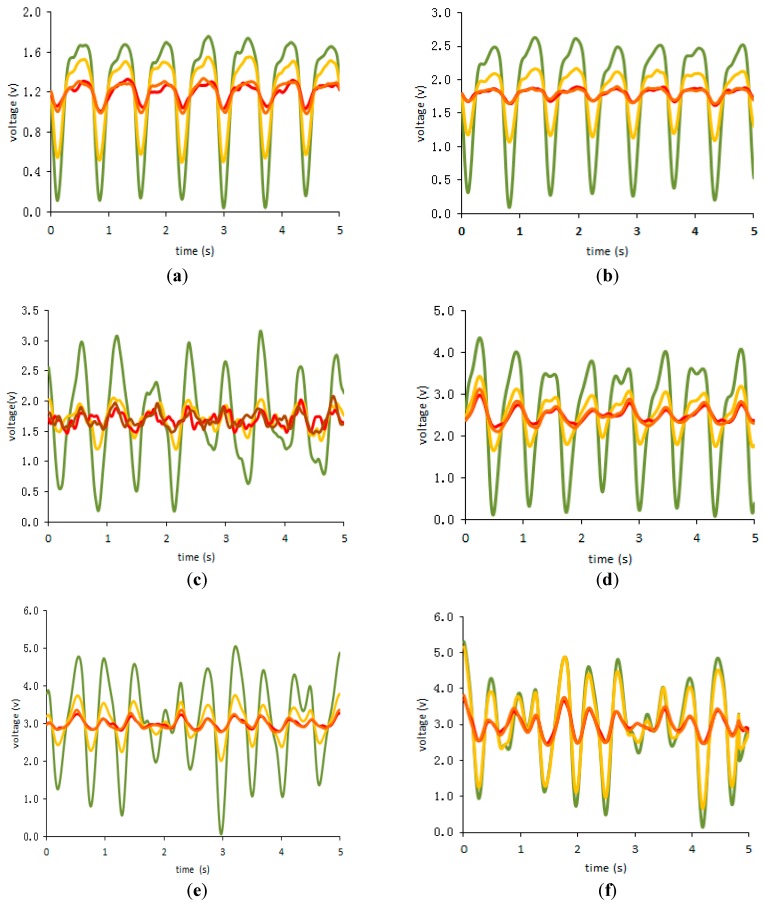
(**a**) resting without auto adaption; (**b**) resting with auto adaption; (**c**) walking without auto adaption (3 km/h); (**d**) walking with auto adaption (3 km/h); (**e**) jogging without auto adaption (6 km/h); (**f**) jogging with auto adaption (6 km/h). The green line stands for the response from the green LED illumination; the orange line stands for yellow LED illumination; the red line stands for red LED illumination; the brown line stands for the peak value illumination.

Figure 10 presents signals from one subject before and after auto adaption gain for different exercises, i.e., resting, walking and jogging.

## 4. Discussion

The outcome achieved from the implementation of the designated experimental protocol has demonstrated the OEPS, with its associated electronics and algorithms. This is also able to meet the specified skin type through comparison between commercial three-lead resting ECG and the OEPS developed in the lab. The optimal illumination to a designated skin type can be selected by a desired spectral wavelength from 525 nm to 870 nm, together with four channels with 16 LEDs, through an auto-adaptive adjustment. Undoubtedly, multi-wavelength illuminations, for instance having more LEDs attached to the OEPS, could improve the performance of opto-physiological monitoring. A better performance microcontroller, i.e., dsPIC33FJ256MC710, could be ideal to control these multiplexed LED drivers and to demultiplex the signals captured from the PD. However, this could increase the complexity of the OEPS system. Thus, a better solution to optimize the trade-off between performance and cost is developing a forthcoming OEPS architecture design. Figure 7 presents the HR measurement between the OEPS and the three-lead resting ECG during resting in a higher correlation (*r:* 0.982). As depicted in the Bland–Altman plot in Figure 8, the HR outputs recorded at resting are most likely to be in the acceptable range of *B ±* 1.96 *SD* in the HR difference between the three-lead resting ECG and the OEPS. From Figure 7 and Figure 8 it can be derived that the HR measurements obtained from the commercial ECG device and the OEPS are not only compatible but satisfactory as well [18].

As presented in Figure 9, the HRs among the different five types of skin during the four types of exercises, were measured by means of the OEPS. The largest value of SDR during resting and walking is 7.2 bpm. While jogging and running, the HRs are fluctuated significantly and the largest SDR reaches up to 21.9 bpm. The main reasons for the increase in the SDR value may be caused by excessive strain of the OEPS sensor applied on the subject palm as well as the electrical noise from the PD during the exercises. In addition, the HRs on different people could be varied due to individual blood circulations. The two-way ANOVA showed that the activities have a notable factor because *p*_1_ ≈ 0 (*F* = 323.2) which is less than *p* = 0.01, whilst the different types of skin did not have a significant factor because *p*_2_ = 0.09 (*F* = 3.0) as it is more than *p* = 0.05.

Referring to the auto adaption effect shown in Figure 10a–f, the intensities of the PPG signals appear to be higher with the auto gain applied compared to those without the auto gain. As depicted in Figure 10, the auto gain is able to improve the quality of the signal-to-noise ratio (SNR) resulting in more precise and easy-to-obtain HR measurements. Meanwhile, starting from the resting exercise to the jogging one, the intensities of the PPG signals became higher over time. As the intensity of the physical activity in the jogging exercise increased, the intensity of the PPG signals corresponding to the illuminations of yellow, red and infrared LEDs was also increased significantly, whilst the intensity of the PPG signals from the green illumination remained stable. However, the motion artifact occurred predominantly with the increase of the physical activity resulting in imprecise HR measurements contrary to resting or moderate activity.

Fallow, B.A. et al. [6] mentioned that four-different-wavelength illumination was used in the assessment of HR detection (Blue 470 nm, Green 520 nm, Red 630 nm, and Infrared 870 nm). The results showed that green illumination has greater modulation than the other wavelength illuminations regardless of skin types. As a matter of fact, the same conclusion was reached in the utilization of the OEPS as shown in Figure 10. Thus, the HRs were mainly attained by the green wavelength illumination in the OEPS.

## 5. Conclusions

A multi-wavelength opto-electronic patch sensor (OEPS), through the implementation of a designated exercise protocol, has been proved to effectively detect physiological signals against skin pigments. The auto adaptation module has been realized to optimally select a suitable illumination for the right type of skin. The study shows the approach of acquiring and processing physiological signals that could be effectively applied in real time, such as sending signals wirelessly to a PC monitor or a server on a receiving end user. With a comparison measurement, the compatibility of HRs between the ECG and the OEPS was proven to be in an acceptable range of the HR difference using the three-lead ECG and the OEPS. Specifically, the outcome of the experimental protocol, with the participation of 33 healthy male subjects, demonstrates the OEPS ability to measure HR effectively regardless of the skin types during various physical activities. The OEPS, with multi-wavelength illuminations, could be extended to the scope of sport physiological monitoring application, and could be applied in various ethnic groups for cost-effective health monitoring and assessment. Furthermore, the OEPS could be consolidated to already available wearable and smart devices for in-line and real-time monitoring and assessment. Nevertheless, several challenges such as different OEPS measurement locations on the skin with relevantly rich peripheral blood vessels, optimum wavelength illuminations, motion artifact, and even electronic noises, are to be addressed in the upcoming studies.

## Figures and Tables

**Table 1:** Human skin types and associated region [8].

Type	Color/Description	Sun-burning	VLCS	Region/Area
I & II	Light/White	Often/Usually	1–10	Caucasus/Europe
III	Medium, white to light brown	Rarely	11–15	Asia
IV	Olive, moderate brown	Rarely	16–21	Middle East
V	Brown, dark brown	Very rarely	22–28	Africa

**Table 2:** Approximate penetration depth of optical radiation in skin tissue [13].

Wavelength (nm)	500	600	700	800	1000	1200
Depth (µm)	230	550	750	1200	1600	2200

**Table 3:** Ranges of Average value and standard deviation of human skin types across activities.

Skin Type	Resting	Walking: 3 km/h	Jogging: 6 km/h	Running: 9 km/h
I & II (n = 11)	62~92	77~133	100~139	104~170
	(1.0~4.3)	(1.7~5.4)	(4.2~11.9)	(4.6~12.8)
III (n = 10)	57~94	71~132	87~144	113~170
	(1.3~3.4)	(1.3~7.2)	(1.9~10.2)	(6.1~20.8)
IV (n = 7)	62~93	78~133	99~147	98~163
	(1.7~5.1)	(1.3~6.3)	(2.4~8.1)	(3.6~21.9 *)
V (n = 5)	73~90	106~116	122~123	131~154
	(3.7~5.9)	(1.9~4.1)	(5.4~10.4)	(3.1~5.3)

* The largest value of SDR in all activities; n is number of subjects

**Table 4:** Average value of HR and standard deviation with four skin types under exercises (n is number of subjects).

Skin Type	Resting	Walking: 3 km/h	Jogging: 6 km/h	Running: 9 km/h
I & II (n = 11)	74(2.2)	93(3.5)	112(7.1)	124(8.8)
III (n = 10)	72(2.3)	96(3.1)	117(5.0)	136(13.4)
IV (n = 7)	74(2.6)	97(4.9)	115(5.1)	134(9.4)
V (n = 5)	77(4.8)	100(3.0)	116(7.9)	131(4.2)
